# Intestinal Exposure to Ceftiofur and Cefquinome after Intramuscular Treatment and the Impact of Ceftiofur on the Pig Fecal Microbiome and Resistome

**DOI:** 10.3390/antibiotics11030342

**Published:** 2022-03-04

**Authors:** Sofie Rutjens, Nick Vereecke, Ward De Spiegelaere, Siska Croubels, Mathias Devreese

**Affiliations:** 1 Department of Pathobiology, Pharmacology and Zoological Medicine, Faculty of Veterinary Medicine, Ghent University, 9820 Merelbeke, Belgium; sofie.rutjens@ugent.be (S.R.); siska.croubels@ugent.be (S.C.); 2 PathoSense BV, 2500 Lier, Belgium; nick.vereecke@pathosense.com; 3 Department of Translational Physiology, Infectiology and Public Health, Laboratory of Virology, Faculty of Veterinary Medicine, Ghent University, 9820 Merelbeke, Belgium; 4 Department of Morphology, Imaging, Orthopedics, Rehabilitation and Nutrition, Faculty of Veterinary Medicine, Ghent University, 9820 Merelbeke, Belgium; ward.despiegelaere@ugent.be

**Keywords:** intramuscular administration, cephalosporins, UHPLC-MS/MS, gut and fecal excretion, swine, fecal microbiome, resistome

## Abstract

Optimization of antimicrobial treatment during a bacterial infection in livestock requires in-depth knowledge of the impact of antimicrobial therapy on the pathogen and commensal microbiota. Once administered antimicrobials and/or their metabolites are excreted either by the kidneys through urine and/or by the intestinal tract through feces, causing antimicrobial pressure and possibly the emergence of resistance in the gastro-intestinal tract. So far, the excretion of ceftiofur and cefquinome in the intestinal tract of pigs has not been described. The objective of this study was to investigate the excretion of ceftiofur and cefquinome in the different segments of the gut and feces after intramuscular administration. Therefore, 16 pigs were treated either with ceftiofur (*n* = 8) or cefquinome (*n* = 8), and feces were collected during the entire treatment period. The presence of ceftiofur and desfuroylceftiofuracetamide or cefquinome were quantified via liquid chromatography–tandem mass spectrometry. At the end of the treatment, pigs were euthanized, and samples from the duodenum, jejunum, ileum and cecum were analyzed. In feces, no active antimicrobial residues could be measured, except for one ceftiofur-treated pig. In the gut segments, the concentration of both antimicrobials increased from duodenum toward the ileum, with a maximum in the ileum (187.8 ± 101.7 ng·g^−1^ ceftiofur-related residues, 57.8 ± 37.5 ng·g^−1^ cefquinome) and sharply decreased in the cecum (below the limit of quantification for ceftiofur-related residues, 6.4 ± 4.2 ng·g^−1^ cefquinome). Additionally, long-read Nanopore sequencing and targeted quantitative polymerase chain reaction (qPCR) were performed in an attempt to clarify the discrepancy in fecal excretion of ceftiofur-related residues between pigs. In general, there was an increase in *Prevotella, Bacteroides* and *Faecalibacterium* and a decrease in *Escherichia* and *Clostridium* after ceftiofur administration (*q*-value < 0.05). The sequencing and qPCR could not provide an explanation for the unexpected excretion of ceftiofur-related residues in one pig out of eight. Overall, this study provides valuable information on the gut excretion of parenteral administered ceftiofur and cefquinome.

## 1. Introduction

Since their introduction, antimicrobials have been commonly used for the treatment, control and prevention of infectious diseases in both human and animals. Although they were developed to target pathogenic bacteria, many antimicrobials also reach the gastro-intestinal tract and may affect the gut microbial community [1,2,3,4,5]. It has been reported that even a transient alteration can result in long-term consequences for the host, e.g., impacted growth and altered immune functions [6,7,8,9,10,11]. Numerous studies concluded that low levels of antimicrobials or derivatives impact the gut microbial ecosystem in such way that increased growth rates in weaned piglets have been measured [12,13,14,15,16]. Antimicrobials not only have an impact on shifts of the host gut microbiome composition, but they also cause the selection and emergence of bacterial strains harboring antimicrobial resistance genes [2,17,18,19,20]. This is of particular concern in the gut, since it is considered a reservoir of antimicrobial resistance genes [21]. Multiple studies concluded that the administration of antimicrobials leads to the proliferation of bacterial strains harboring antimicrobial resistance genes conferring resistance toward the administered drug and other antimicrobials. A study by Römer et al. (2017) detected a substantial reduction in the amount of susceptible intestinal *E. coli*, the indicator for Gram-negative microbiota, after two periods of parenteral administration of enrofloxacin with an interval of 2 weeks to 5–6-week-old pigs. While resistant *E. coli* were detected in only two animals after the first treatment period, all pigs harbored resistant *E. coli* after the second treatment period [18]. In another study, a similar result was obtained after a single treatment with ceftiofur. However, in this study, the prevalence of resistant *E. coli* was only observed at 48 h post-administration, followed by a gradual decrease in these levels after treatment [20]. Even though the presence of resistant *E. coli* was temporary, the feces of these pigs were thought to become a reservoir of resistant bacteria and antimicrobial residues.

So far, there is only little information on the intestinal and fecal concentrations of parenterally injected cephalosporins [22,23]. The third and fourth generation cephalosporins, in particular ceftiofur and cefquinome, are developed for parenteral therapeutic use in veterinary medicine against bacterial infections in livestock caused by various Gram-positive and Gram-negative organisms [24,25,26,27,28,29,30]. Evidence for exposure of the gut microbial community to these antimicrobials and/or their active residues exists in pigs where treatment with parenteral ceftiofur or cefquinome caused the development of resistant gut bacteria [20,31,32,33,34]. After excretion, these antimicrobial residues not only caused the selection and emergence of antimicrobial resistance genes, but the residues themselves can also spread in the environment. However, no data are available on the concentration levels reached in the intestinal tract and the evolution of the gut exposure throughout the different segments. This is especially important, as β-lactam antibiotics are generally accepted to be renally excreted. Therefore, determining the concentration of these antimicrobials and their metabolites in the gut and feces will provide valuable information on the impact of ceftiofur and cefquinome [35,36].

The main objective of this study was to investigate the gut and fecal excretion of two cephalosporins, ceftiofur and cefquinome, in 8-week-old pigs. After administration, ceftiofur is metabolized rapidly to its active residue, desfuroylceftiofur. Since desfuroylceftiofur is known to be unstable, an optimized sampling protocol was followed, and during analysis, the combinations of ceftiofur and its related residues (e.g., desfuroylceftiofur) were derivatized and measured as desfuroylceftiofuracetamide [37]. The second objective of this study was to explore the impact of ceftiofur treatment on the fecal microbiome and its resistome. To investigate this, long-read nanopore shotgun sequencing and a targeted qPCR assay were conducted on the pre- and post-treated fecal samples of the ceftiofur-treated pigs.

## 2. Results and Discussion

### 2.1. Animal Experiment

During antimicrobial treatment, no side effects were observed. All animals remained clinically healthy during the entire experiment.

### 2.2. Ceftiofur, Ceftiofur-Related Metabolites and Cefquinome: Plasma Concentrations

**Figure 1:**
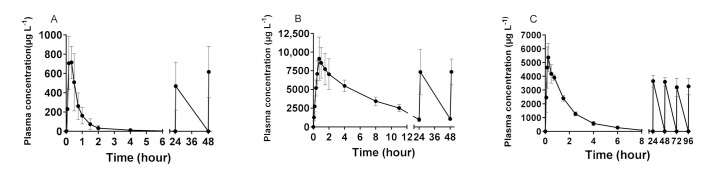
(**A**) Mean plasma concentrations (±standard deviation; *n* = 8) of ceftiofur after the first administration (0–4 h) and for the timepoints right before and at the time of maximum concentration (T_max_) of every repeated administration during the entire treatment period (0–49 h) with intramuscular administration of 3 mg·kg^−1^ body weight. The bolus was administered once daily for 3 consecutive days. (**B**) Mean plasma concentrations (±standard deviation; *n* = 8) of ceftiofur and ceftiofur-related residues, measured as desfuroylceftiofuracetamide, after the first administration of ceftiofur (0–11 h) and for the timepoints right before and at the T_max_ of every repeated administration during the entire treatment period (0–49 h). (**C**) Mean plasma concentrations (±standard deviation; *n* = 8) of cefquinome after the first administration (0–6 h) and for the timepoints right before and at the T_max_ of every repeated administration during the entire treatment period (0–96.5 h) with intramuscular administration of 2 mg·kg^−1^ body weight. The bolus was administered once daily for 5 consecutive days.

The average plasma concentration for ceftiofur, desfuroylceftiofuracetamide and cefquinome is depicted in Figure 1. The calculated plasma pharmacokinetic (PK) parameters can be found in Table 1. Interestingly, ceftiofur reaches its peak concentration at 20 min post-administration, suggesting an efficient absorption from the muscle tissue to the circulatory system. For all analytes, the data suggest no accumulation after repeated administration. For cefquinome, the T_max_ used to determine the sampling points for the repeated administrations differed slightly from the T_max_ calculated here (0.5 h vs. 0.3 h), explaining the lower measured maximum concentration after repeated administration. The calculated plasma PK parameters of desfuroylceftiofuracetamide and cefquinome are in line with those reported by other publications. [38,39].

### 2.3. Ceftiofur, Ceftiofur-Related Metabolites and Cefquinome: Intestinal and Fecal Concentrations

The average concentration (±standard deviation) of the ceftiofur-related residues, measured as desfuroylceftiofuracetamide, and cefquinome in the different gut segments at 11 h after last administration is summarized in Table 2.

In both groups, the concentration increased toward the ileum, followed by a sharp decrease in the cecum. This initial increase can be explained by a combination of analyte excretion and resorption of water during the transit in the duodenal, jejunal and ileal gut segments. The decrease in the more distal part of the gut suggests a rapid degradation once the antimicrobials reach the cecum. A possible explanation for this cecal degradation is the presence of a higher number of bacteria (10^11^–10^12^ CFU·mL^−1^) in the large intestine of pigs compared to the relatively low numbers in the small intestine (10^1^–10^7^ CFU·mL^−1^) [40,41]. Furthermore, a difference in microbial composition between the segments has been described [42,43,44,45,46]. A study by Isaacson (2012) showed that the gut microbiome at phylum level in the ileum of 11-week-old pigs contained more than 95% Firmicutes, while the cecum and colon were dominated by an approximately equal amount of Firmicutes and Bacteroidetes [42]. Similar results were obtained in the study by Dowd (2008), where several members of Firmicutes dominated the ileum of weaned piglets [43]. In a study by Looft (2014), the ileum of 3-month-old pigs was dominated by Anaerobacter and Turicibacter, while the cecum contained Prevotella, Oscillibacter and Succinivibrio, which is in agreement with the above studies [44]. Of these genera, Prevotella is known to carry beta-lactamase encoding genes; its absence in the ileum and presence in the cecum could explain the degradation of these antimicrobials in the cecum [47,48]. Besides Prevotella, Bacteroides also possesses a wide array of these beta-lactamase encoding genes [49]. In general, the administration of ceftiofur results in significantly higher concentrations in the duodenum and ileum in comparison to cefquinome. This is in line with the higher administered dose (3 mg·kg^−1^ vs. 2 mg·kg^−1^ bodyweight (BW)). However, normalized to dose, ceftiofur still reaches higher concentrations in the different gut segments. Nevertheless, cefquinome is still present in the cecum, suggesting a less efficient degradation. The impact of this concentration difference on the gut microbiota and the emergence of antimicrobial resistance genes will be investigated in a follow-up study.

**Figure 2:**
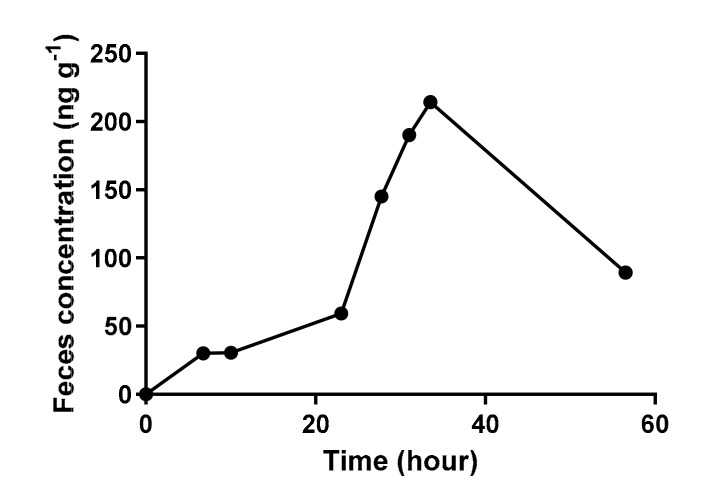
Fecal concentrations of ceftiofur-related residues, measured as desfuroylceftiofuracetamide, in fecal samples of one female pig (pig n° 2) following intramuscular administration of 3 mg·kg^−1^ body weight of ceftiofur.

Ceftiofur, ceftiofur-related residues and cefquinome concentrations in fecal samples were below the limit of quantification (LOQ) of 5 ng·g^−1^ for most pigs. However, in the ceftiofur group, all fecal samples of one female pig (pig n° 2) contained measurable amounts of ceftiofur-related residues, measured as desfuroylceftiofuracetamide (Figure 2). In a recently published study by Gaire (2021) on finisher pigs, ceftiofur-related residues could be detected in the feces of all treated pigs (*n* = 25), while in our study, the feces of only one out of eight pigs contained residues [23]. A possible explanation could be that the higher dose of ceftiofur received by the finisher pigs (4.4 mg·kg^−1^ BW vs. 3 mg·kg^−1^ BW) resulted in a higher concentration in the gut. Another explanation could be the varying composition of the gut microbiota between these two groups of pigs either due to age (4 months old vs. 8 weeks old), genetics, diet and/or environmental differences [50,51,52,53,54,55,56,57]. Kim (2011) described a shift in composition of the gut microbiota of 10-week-old pigs until the pigs were 22 weeks old [51]. In their study, Prevotella was the most abundant genus (30%) of all classifiable bacteria in the microbiome of 10-week-old pigs. However, at the age of 22 weeks, Prevotella comprised only 3.5–4.0% of the gut bacteria. Another interesting study analyzed the pig fecal microbiota of three 15-week-old purebred pig lines (Duroc, Landrace and Yorkshire) [52]. These pigs were fed the same feed and were housed in environmentally controlled facilities. The results showed that at the genus level, a difference was demonstrated between pigs from different breeds. The study by Gaire (2021) did not mention which breed the pigs belonged to. Interestingly, the concentrations of desfuroylceftiofuracetamide in the different gut segments of pig n° 2 were in the same range as in the other pigs, suggesting a slower degradation during gut transit. A varying constitution of the fecal microbiota between the individual pigs could possibly explain this slower degradation.

### 2.4. Impact of Ceftiofur on the Gut Microbiome of Pigs

**Figure 3:**
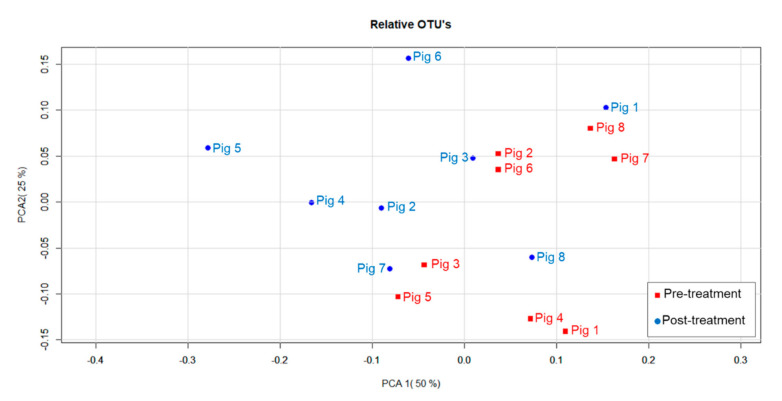
Principal coordinate analysis of fecal microbiomes. Relative total number of operational taxonomic units (OTUs) in the fecal microbiome of each animal (*n* = 8) pre-treatment (■) and post-treatment (●) were analyzed via principal coordinate analysis using Bray–Curtis dissimilarity matrix (daily intramuscular administration of 3 mg·kg^−1^ body weight of ceftiofur for 3 consecutive days).

A principal coordinate analysis (PCoA), using the Bray–Curtis dissimilarity index, was performed to compare pre-treatment microbiome composition to post-treatment (Figure 3). The distance between points indicates the difference in microbiome composition, with points closer together being more similar. Permutational multivariate analysis of variance using distance matrices (adonis) indicates a statistically significant (*p* = 0.04795) variation of 16% that could be explained by the impact of treatment and time. Since there are numerous factors that shape the microbiome, this suggests a significant alteration caused by ceftiofur treatment and time [58]. In a more determinative follow-up study, samples of a control group will be analyzed to filter out the impact of time on the gut microbiome.

**Figure 4:**
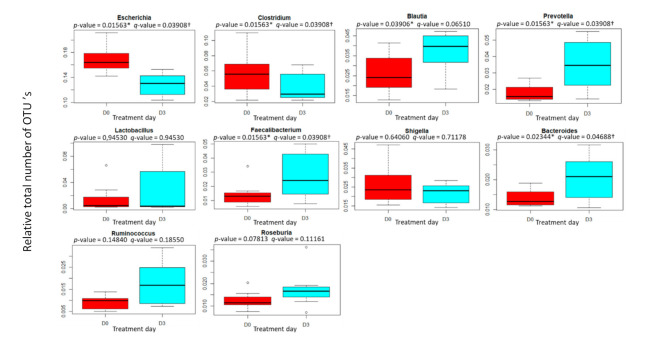
Box-and-whisker plots showing the mean (*n* = 8) relative total number of OTUs of the top 10 genera pre-treatment (D0) and post-treatment (D3), i.e., 9 h after the last dose of the daily intramuscular administration of 3 mg·kg^−1^ body weight of ceftiofur for 3 consecutive days. Boxes represent the interquartile ranges, the median is depicted by the lines inside the boxes, the whiskers represent the 25% quantile or the 75% quantile plus 1.5 times the interquartile range. Significance codes: ‘*’ *p*-value < 0.05, ‘†’ *q*-value < 0.05 (false-discovery-rate-corrected *p*-value).

Long-read sequencing resulted in 509,622 (±165,845) reads, an N50 read length of 2805 (±783) nucleotides and an overall sequencing throughput of 754,776,826 (±310,359,273) bases per sample, allowing for genus level classification. An overview of the identified bacterial genera is shown in Figure 4 (and Supplementary Figure S1). The graphs are based on the relative total number of OTUs of the top 10 genera in each sample to allow a comparison of genera composition before and after treatment. Ceftiofur administration resulted in a reduction in certain genera and an overgrowth of others. As previously described by Fleury (2015), ceftiofur caused a significant decrease in relative Escherichia abundance (original *p*-value = 0.01563, *q*-value = 0.03908) [59]. However, in the study of Fleury (2015), this was only observed during the first days after a single injection. Approximately three weeks after the single intramuscular injection of ceftiofur crystalline free acid (5 mg·kg^−1^), the microbial population returned close to its initial level. This decrease was also observed in dairy cattle after the administration of ceftiofur (2.2 mg·kg^−1^, intramuscularly, once per day for 5 days) [60]. In the present study, the last sample was taken approximately 3.5 days after the first administration, preventing the observation that normal levels of Escherichia could be returned. In another study, a decrease in E. coli abundance was also observed after the administration of amoxicillin and the β-lactamase inhibitor, clavulanic acid, to weaning pigs [61]. In contrast to the study by Zeineldin (2018), a significant increase (original *p*-value = 0.01563, *q*-value = 0.03908) in Prevotella was observed in this current study. Zeineldin did report an increase in Prevotella on the first day after the administration of ceftiofur hydrochloride, albeit not significant. Afterward, the proportion of Prevotella remained close to its initial level [4]. In the study by Zeineldin, an increase in Prevotella was not observed after the administration of ceftiofur crystalline free acid, indicating that dosage and pharmaceutical form (i.e., slow release) could be responsible for the different results. The observed increase in Prevotella in the current study might also be caused by its potential to carry resistance genes against β-lactam antibiotics [62,63]. Furthermore, in that same study by Zeineldin, a significant increase in the proportion of Blautia was noticed in the ceftiofur hydrochloride group, 7 days after the start of the experiment. This is in line with the results of the current study (original *p*-value = 0.03906, *q*-value = 0.06510), albeit that the increase observed here was no longer significant after false discovery rate (FDR) correction. However, it might be that sampling at 7 days post-treatment instead of 3 days would have resulted in a *q*-value < 0.05, as observed by Zeineldin. So far, the increase in relative numbers of Bacteroides and the decrease in relative numbers of Clostridium in the gut microbiome of pigs after ceftiofur treatment has not been described before. One study did report these changes in the microbiota of dairy cattle after intramuscular ceftiofur administration of 2 mg·kg^−1^ body weight [64]. Interestingly, the relative abundance of Faecalibacterium increased after treatment (original *p*-value = 0.01563, *q*-value = 0.03908). This increase has only been reported before in the microbiome of calves after being fed with a low-concentration cocktail of antibiotics (penicillin, streptomycin, tetracycline and ceftiofur) [65]. A follow-up study will confirm whether these changes in pigs were due to the ceftiofur treatment or whether they were due to coincidence. No significant changes (*q*-value < 0.05) were observed in the current study for any of the other observed genera.

### 2.5. Impact of Ceftiofur on the Gut Resistome of Pigs

**Figure 5:**
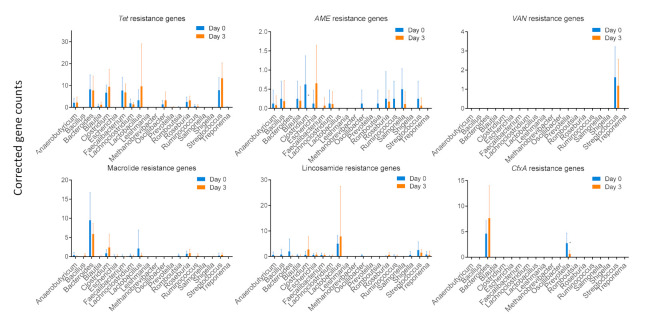
Bar plots (+standard deviation), showing the mean (*n* = 8) resistance gene counts corrected to the relative (pre-treatment to post-treatment, i.e., 9 h after the last dose of the daily intramuscular administration of 3 mg·kg^−1^ body weight of ceftiofur for 3 consecutive days) base counts of the top 20 genera. D0 = day 0, D3 = day 3. Tet = tetracycline, AME = aminoglycoside-modifying enzyme, Van = vancomycine, CfxA = beta-lactamase. Significance codes: ‘*’ *p*-value < 0.05.

**Figure 6:**
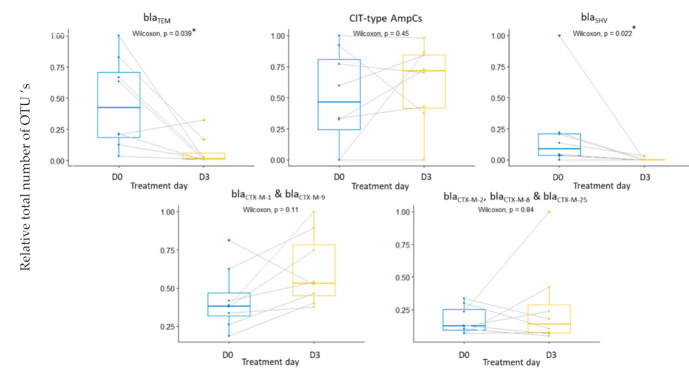
Box-and-whisker plots showing the mean (*n* = 8) relative number of selected resistance genes pre-treatment (D0) and post-treatment (D3), i.e., 9 h after the last dose of the daily intramuscular administration of 3 mg·kg^−1^ body weight of ceftiofur for 3 consecutive days. Boxes represent the interquartile ranges, the median is depicted by the lines inside the boxes, the whiskers represent the 25% quantile or the 75% quantile plus 1.5 times the interquartile range. Significance codes: ‘*’ *p*-value < 0.05.

Figure 5 depicts an overview of the impact of the ceftiofur treatment on the base-corrected counts of recovered resistance genes. To the authors’ knowledge, there are no non-targeted sequencing data available of the impact of beta-lactam antibiotics on the gut resistome of pigs. These data provide interesting information on the collateral effect of the intramuscular administration of ceftiofur. The proliferation of resistant endogenous enterobacteria after ceftiofur administration may result in future infections that are hard to treat or may even be untreatable. Furthermore, the gut microbiota may act as a reservoir for these resistance genes [66]. The antibiotic resistance genes could disseminate between bacterial strains within the microbiome, between individual pigs, between the pigs and farmers, and to the environment [67,68,69]. More information on the data depicted in Figure 5 can be found in the supplementary files (Table S1). To assure no extended-spectrum-beta-lactamase genes were overlooked during the shotgun sequencing, as for lower abundant bacterial species, a targeted qPCR assay was used to check for *bla*TEM, CIT-type AmpCs, *bla*SHV, *bla*CTX-M-1, *bla*CTX-M-2, *bla*CTX-M-8, *bla*CTX-M-9 and *bla*CTX-M-25 (Figure 6). The qPCR results showed a statistically significant decline in *bla*TEM genes of the overall fecal samples. The qPCR data did not show an increase in CTX-M-1 and CTX-M-9 genes. Nevertheless, a significant increase in CTX-M-1 producing E. coli selected from fecal samples of pigs treated with ceftiofur has been observed before [32]. In a study by Cavaco (2008), this CTX-M-1 increase was detected on day 4, i.e., one day after treatment cessation. In contrast, the samples of the current study were taken 9 h after the last dose, possibly explaining the absence of significant results. For Prevotella, there was a significant decrease in associated CfxA genes (*p*-value = 0.0264) (Figure 5). This is in contrast to the increase in relative abundance of Prevotella (Figure 4) and suggests no beneficial effects of CfxA genes on Prevotella. Since CfxA genes encode for beta-lactamase enzymes, special attention was paid to the CfxA genes recovered on Bacteroides and Prevotella.

**Figure 7:**
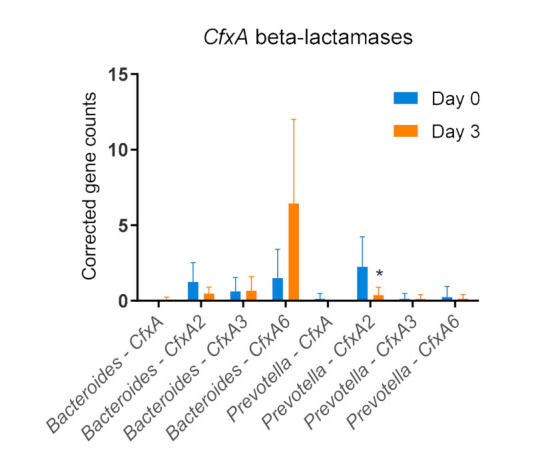
Bar plots (+standard deviation), showing the mean CfxA resistance gene counts on Bacteroides and Prevotella before and after treatment, i.e., 9 h after the last dose of the daily intramuscular administration of 3 mg·kg^−1^ body weight of ceftiofur for 3 consecutive days, corrected to the relative (pre-treatment to post-treatment) base counts of Bacteroides and Prevotella. D0 = day 0, D3 = day 3. Significance codes: ‘*’ *p*-value < 0.05.

The breakdown of the different CfxA beta-lactamases (Figure 7) revealed a statistically significant decrease in CfxA2 on Prevotella (*p* = 0.0469) after ceftiofur treatment. CfxA beta-lactamases are genetically closely related and differ only by point mutations [70]. Several studies have determined different kinetic parameters for the variants of these CfxA genes against a number of beta-lactam antimicrobials [71,72]. To date, there have been no data on the hydrolysis efficiency of the different CfxA variants for ceftiofur. The results depicted here suggest a selection of CfxA6 encoding Bacteroides after ceftiofur treatment, albeit not significantly (*p*-value = 0.0744). Future studies will clarify whether there is a beneficial effect of CfxA6 encoding Bacteroides under selective pressure. A significant reduction in CfxA2 can be observed for Prevotella. Although there is a significant increase in the relative number of OTUs of Prevotella (Figure 4), this decrease in CfxA2 genes suggests no remarkable advantageous effects of the presence of the CfxA2 gene. The other CfxA genes on Prevotella, on the other hand, remain at their initial levels.

### 2.6. Pig n° 2

**Figure 8:**
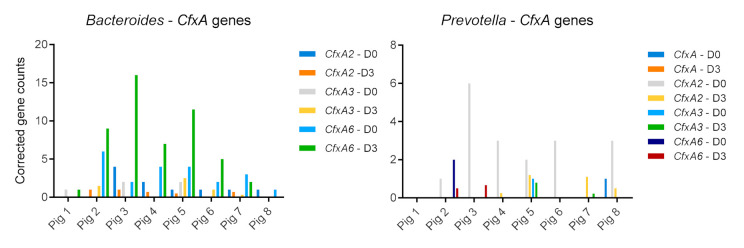
Bar plots showing the CfxA resistance gene counts on Bacteroides and Prevotella per pig, before and after treatment, corrected to the relative (pre-treatment to post-treatment) base counts of Bacteroides and Prevotella. D0 = day 0, D3 = day 3, i.e., 9 h after the last dose of the daily intramuscular administration of 3 mg·kg^−1^ body weight of ceftiofur for 3 consecutive days.

As previously described, one out of eight ceftiofur-treated pigs excreted measurable concentrations of ceftiofur-related metabolites, measured as desfuroylceftiofuractemide, in its feces (see Section 2.3). The plasma pharmacokinetics of this pig showed a slightly faster elimination in plasma for ceftiofur. However, for desfuroylceftiofuracetamide, this more rapid elimination in plasma could not be detected. All other plasma pharmacokinetic properties did not reveal a possible explanation for the presence of desfuroylceftiofuracetamide in feces. The previously described microbiome and resistome results (see Section 2.4 and Section 2.5) could visualize the impact of ceftiofur treatment on the overall microbiome and resistome; however, no clear distinction between pig 2 and the other pigs from the ceftiofur group could be made. Figure 8 shows a breakdown of the CfxA genes for each pig before and after treatment. Overall, there is a clear increase in CfxA6 genes on Bacteroides and a decrease in CfxA2 genes on Prevotella in most pigs, as described before. The data suggest a higher hydrolysis activity in the feces of pig 2, considering the higher relative number of CfxA6 genes on both Bacteroides and Prevotella before treatment. This contradicts the measured ceftiofur-residue concentrations. However, it has been previously reported that variable expression and even misexpression of these genes is possible [70,73]. To date, it remains unclear what mechanism is at the basis of the excretion discrepancy between the pigs.

## 3. Methods

### 3.1. Standards and Chemicals

Ceftiofur, cefquinome sodium and cefotaxime were purchased from Sigma-Aldrich (Overijse, Belgium). Ceftiofur-d3 and cefquinome-d7, used as isotopically labeled internal standard (IS), were purchased from Toronto Research Chemicals (North York, Canada). Tazobactam was purchased from Sigma-Aldrich (Overijse, Belgium). Methanol and acetonitrile were of UHPLC grade and were obtained from Fisher Scientific (Erembodegem, Belgium). Water was obtained from a Milli-Q water purification system. Products and solvents used for preparation of buffer solutions and for extraction (trichloroacetic acid or TCA, sodium acetate, formic acid or FA, acetic acid, potassium di-hydrogen phosphate or KH_2_PO_4_, sodium hydroxide or NaOH, potassium hydroxide or KOH, potassium chloride, dimethylsulfoxide or DMSO, tris(hydroxymethyl)aminomethane or TRIS, ammonium formate, hydrochloric acid or HCl, sodium-tetraborate, dithioerythritol or DTE, iodoacetamide) were HPLC grade and obtained from Merck (Darmstadt, Germany) and Sigma-Aldrich (Overijse, Belgium).

### 3.2. Preparation of Solutions

For the analysis of ceftiofur and cefquinome in plasma, a 1 M acetate buffer pH 5 was prepared by dissolving 47.63 g of sodium acetate in 400 mL of ultrapure water and adding 12.87 mL acetic acid. Thereafter, the volume was completed further to 500 mL with ultrapure water. The buffer was stored at 2–8 °C. For the derivatization of ceftiofur and ceftiofur-related residues, a hydrolysis solution for derivatization was prepared by dissolving 3.7 g of potassium chloride, 19.0 g sodium-tetraborate and 4.0 g of dithioerythritol in 1000 mL of ultrapure water. The hydrolysis solution was stored for maximum 5 days at 2–8 °C. The phosphate buffer (0.025 M, pH 7.0) was prepared by adding 3.4 g of KH_2_PO_4_ to 700 mL of water and adjusting the pH to 7.0 with concentrated potassium hydroxide. Water was added to obtain a total volume of 1000 mL. Thereafter, 14 g of iodoacetamide was dissolved in 100 mL of this phosphate buffer to obtain a 14% (*w/v*) iodoacetamide solution and stored at 2–8 °C and shielded from light. A 1 mg·mL^−1^ stock solution of tazobactam was prepared in water. This stock solution was diluted with UHPLC-water to a concentration of 8000 µg·L^−1^ and stored at ≤−70 °C. A separate stock solution of 1 mg·mL^−1^ of ceftiofur and ceftiofur-d3 was prepared in DMSO. The cefquinome stock solution of 1 mg·mL^−1^ was prepared in UHPLC-water. The cefquinome-d7 stock solution of 1 mg·mL^−1^ was prepared in methanol/DMSO 10/1 (*v/v*). The stock solutions were divided in Eppendorf cups (Novolab, Geraardsbergen, Belgium) and stored at ≤−70 °C.

### 3.3. Animals and Test Article Administration

A group of 16 healthy, stress-resistant, 8-week-old pigs (8 male, 8 female, Landrace × Large White × Maximus, Seghers Hybrid^®^, Wuustwezel, Belgium) of 17.4 kg ± 1.1 kg BW, were group housed in standard pig stables with ad libitum access to water and feed (Biggispeen Premium^®^, Aveve, Leuven, Belgium). After a 4-day acclimatization period, the pigs were gender-based, randomly allocated to the different antimicrobial treatment groups. Prior to the experiment, the animals were never exposed to antimicrobial drugs. The pigs were housed individually to avoid contamination and coprophagy between different pigs.

Ceftiofur was administered (4 male, 4 female pigs) as an intramuscular injection at a dose of 3 mg·kg^−1^ body weight at the same timepoint on three consecutive days, in accordance with the leaflet (Excenel flow^®^, Zoetis, Louvain-la-Neuve, Belgium). Blood was sampled via the jugular vein into K_3_EDTA collection tubes (Vacutest^®^, Piove die Sacco, Kima, Italy) at 0, 5, 10, 20, 30, 45 and 60 min, and at 1.5, 2, 4, 8, 11 and 24 h after the first administration and at 20 min, 1 h and 24 h after the second and third administration. After sampling, all blood samples were centrifuged at 2851 g for 10 min at 4 °C. Plasma was stored at ≤ −70 °C until analysis. Feces were sampled in a sterile plastic cup after non-invasive rectal stimulation before the administration of ceftiofur and at 4, 6.5, 10, 23, 28, 31, 33.5, 46.5, 51, 54 and 57 h after the start of the treatment. It has been previously observed that the fecal microbiota can rapidly degrade the β-lactam ring of ceftiofur, its related metabolites and cefquinome [37,74]. Therefore, immediately after sampling, 500 µL of a tazobactam solution (8 mg·L^−1^) in water was added to an aliquot of 0.5 g sampled feces. The addition of tazobactam ensures the preservation of the exact concentration of the antimicrobial, resulting in a more accurate representation of the antimicrobial concentration. The fecal samples were stored within 30 min post-sampling at ≤−70 °C until analysis.

The second group (4 male, 4 female pigs) received an intramuscular dose of cefquinome at 2 mg·kg^−1^ body weight at the same timepoint on five consecutive days, in accordance with the leaflet (Ceffect^®^, Emdoka, Hoogstraten, Belgium). Blood was sampled via the jugular vein into K_3_EDTA collection tubes (Vacutest^®^, Piove die Sacco, Kima, Italy) at 0, 5, 10, 15, 30 and 45 min, and at 1.5, 2.5, 4, 6, 8 and 24 h after the first administration, and at 30 min and 24 h after the second, third, fourth and fifth administration. After sampling, all blood samples were centrifuged at 2851× *g* for 10 min at 4 °C. Plasma was stored at ≤−70 °C until analysis. Feces were sampled as for the ceftiofur treatment at 4, 7, 10, 23, 27, 30, 33, 47, 52, 55, 58, 71, 74, 77.5, 81, 95, 98, 101 and 105 h after the start of the antibacterial treatment. Aliquots were sampled and stored as mentioned above.

After 3 and 5 days of ceftiofur and cefquinome treatment, respectively, the animals were euthanized 11 h after the last administration. First, the pigs were sedated with an injection of 0.3 mg·kg^−1^ bodyweight xylazine (Xyl-M^®^, V.M.D. Vet, Arendonk, Belgium) and 15 mg·kg^−1^ bodyweight tiletamine-zolazepam (Zoletil 100^®^, Virbac, Barneveld, the Netherlands). Final euthanasia was performed by intracardiac injection of sodium pentobarbital 20% (Kela Veterinaria, Sint-Niklaas, Belgium) at 0.06 mg·kg^−1^ bodyweight. After euthanasia, an aliquot of 0.5 g intestinal content from different gut segments (duodenum, jejunum, ileum and cecum) was collected, and 500 μL of the 8000 μg·L^−1^ tazobactam solution in water was immediately added to each sample. The intestinal samples were stored within 30 min post-sampling at ≤−70 °C until analysis.

The study was conducted with the approval of the Ethical Committee of the Faculty of Veterinary Medicine and the Faculty of Bioscience Engineering of Ghent University (approval number EC 2021-16) and was in compliance with the Belgian and European legislation on animal welfare and ethics [75,76].

### 3.4. Ceftiofur, Ceftiofur-Related Metabolites and Cefquinome: Plasma Measurement

Total plasma concentration of ceftiofur, cefquinome and ceftiofur in combination with its metabolites, measured as desfuroylceftiofuracetamide, was quantified using two in-house validated (ultra)high-performance liquid chromatography–tandem mass spectrometry ((U)HPLC-MS/MS) methods.

To determine ceftiofur and cefquinome in plasma samples, matrix-matched calibration curves, ranging from 10 to 1000 µg·L^−1^ for ceftiofur and from 200 to 15,000 µg·L^−1^ for cefquinome, were constructed in ultrapure water. The sample preparation was as follows: to 250 µL of plasma, 25 µL of the internal standard solution, consisting either of 10,000 µg·L^−1^ ceftiofur-d3 or of 10,000 µg·L^−1^ cefquinome-d7, was added. After vortex mixing, 60 µL of a 10% (*w/v*) trichloroacetic acid solution in water was added for deproteinization. The samples were vortex mixed, followed by centrifugation for 10 min (1500*× g* at 4 °C). Afterward, 275 µL of the supernatant was transferred to an Eppendorf cup, and 50 µL of a 5% (*w/v*) tris(hydroxymethyl)aminomethane solution in ultrapure water was added, followed by 37.5 µL of a 1 M acetate buffer pH 5 and 31 µL of acetonitrile. The samples were vortex mixed, whereafter 10 µL was injected into the LC-MS system. The LC-MS/MS system consisted of a Quattro Ultima^®^ triple quadrupole mass spectrometer from Waters (Millford, MA, USA) with Masslynx software (version 4.0) combined with a Waters Alliance 2695 system (Zellik, Belgium). The analytes were separated on a Zorbax Eclipse Plus column (Reversed Phase C18, 100 mm *×* 2.1 mm i.d., dp: 3.5 µm) combined with a guard column of the same type, both from Agilent Technologies (Diegem, Belgium). The mobile phases for chromatographic separation consisted of 0.1% (*v/v*) formic acid and 2 mM ammonium formate in ultrapure water (A) and 0.1% (*v/v*) formic acid in ACN (B). The flow rate was set at 400 µL·min^−1^. The temperature of the autosampler tray was set at 4.0 °C. The following tune parameters were used: capillary voltage, 3.5 kV; cone voltage, 20 V; source temperature, 120 °C; desolvation temperature, 400 °C. The optimal settings for collision energy for fragmentation of the molecular ion (or precursor ion) were 20 eV for ceftiofur and 15 eV for cefquinome. Following transitions, (*m/z*) were used for identification and quantification (*quantification ion, most intense product ion): cefquinome: *m/z* 529.0 > 134.0 *, 396.30; cefquinome-d7: *m/z* 536.4 > 141.0 *; ceftiofur: *m/z* 524.0  >  240.9 *, 126.0; ceftiofur-d3: *m/z* 527.0  >  244.1 *. Validation results of ceftiofur and cefquinome in plasma samples demonstrated a precision and accuracy within ± 8.2% at concentration levels of 10 (LOQ), 100 and 1000 µg·L^−1^ for ceftiofur and within ± 7% at concentration levels of 200 (LOQ), 1000 and 15,000 µg·L^−1^ for cefquinome.

Ceftiofur and ceftiofur-related residues were measured as desfuroylceftiofuracetamide (DFCA) in porcine plasma with a UHPLC-MS/MS method following derivatization. Matrix-matched calibration curves, ranging from 25 to 15,000 µg·L^−1^, were prepared in ultrapure water to quantify the samples. For sample preparation, the following procedure was applied: to 250 µL of plasma, 25 μL of internal standard (10,000 µg·L^−1^ cefotaxime in ultrapure water) was added in a 15 mL falcon tube. The first step of the derivatization consisted of adding 2 mL of the hydrolysis solution to the falcon tube and placing the tubes in a water bath of 50 °C (±5 °C) for 15 min. Afterward, the samples were allowed to cool down at room temperature, and 400 µL of the 14% (*w/v*) iodoacetamide in phosphate-buffered solution was added. The samples were vortex mixed, followed by an incubation of 30 min at room temperature, shielded from light. After derivatization, the samples were centrifuged for 10 min (1500× *g* at 4 °C). A 60 mg, 3 mL Oasis HLB SPE cartridge was conditioned using 1 mL of methanol, followed by 1 mL of ultrapure water. The entire supernatant was applied onto the SPE cartridge. The cartridge was then washed with 1 mL of 5% methanol in ultrapure water. After drying under vacuum for 10 min, desfuroylceftiofuracetamide was eluted using 1 mL of 5% acetic acid in acetonitrile. The eluent was evaporated to dryness (45 °C, N2), and the residue was re-dissolved in 250 µL of 0.005% (*v/v*) formic acid in ultrapure water, vortex mixed for 15 s and transferred to an autosampler vial. A 5-μL aliquot was injected into the UPLC-MS/MS system. The UPLC-MS/MS system consisted of a Quattro Premier XE^®^ triple quadrupole mass spectrometer from Micromass (Manchester, UK), with Mass-lynx software (version 4.0) combined with a Waters Acquity UPLC sample and solvent manager (Milford, MA, USA). Analyte separation was achieved on a Acquity UPLC BEH C18 column (Reversed Phase C18, 50 mm × 2.1 mm i.d., dp: 1.7 µm) combined with a guard column of the same type, both from Waters. The mobile phases for chromatographic separation consisted of 0.005% (*v/v*) formic acid in ultrapure water (A) and ACN (B). The flow rate was set at 300 µL·min^−1^. The temperature of the autosampler tray was set at 10.0 °C. The following tune parameters were used: capillary voltage, 2.5 kV; cone voltage, 35 V; source temperature, 120 °C; desolvation temperature, 250 °C. The optimal settings for collision energy for fragmentation of the molecular ion (or precursor ion) were 22 eV for DFCA and for cefotaxime. The following transitions (*m/z*) were used for identification and quantification (*quantification ion, most intense product ion): DFCA: *m/z* 487.2 > 241.2 *, 167.1 and cefotaxime: *m/z* 456.2 > 324.3 *. Validation demonstrated a precision within ±7.7% and an accuracy within ±4.5% at 25 (LOQ) and 2500 µg·L^−1^.

### 3.5. Antibiotic Fecal Measurement

The fecal concentration of ceftiofur, desfuroylceftiofuracetamide and cefquinome was determined using (U)HPLC-MS/MS methods, as previously described [37]. The LOQ was 5 ng·g^−1^ for ceftiofur, 30 ng·g^−1^ for desfuroylceftiofuracetamide and 5 ng·g^−1^ for cefquinome.

### 3.6. Pharmacokinetic Analysis of Plasma Concentrations

The non-compartmental pharmacokinetic analysis of the plasma concentration–time data was performed using Phoenix^®^ (Pharsight-Certara, Princeton, NJ, USA). Values below the LOQ were excluded. A linear up/log down trapezoidal method was used to calculate the area under the curve (AUC). Several PK parameters were calculated: area under the ∞ h-curve (AUC_0→∞_), area under the 2 h time curve for ceftiofur (AUC_0–2 h_)_,_ area under the 24 h time curve for desfuroylceftiofuracetamide (AUC_0–24 h_), area under the 4 h time curve for cefquinome (AUC_0–4 h_), maximal plasma concentrations of all analytes (C_max_), time of maximum concentration (T_max_), elimination of half-life (T_1/2el_).

### 3.7. Fecal DNA Extraction, Nanopore Genome Sequencing, Metagenomic Analysis and Quantitative Polymerase Chain Reaction

To assess the impact of ceftiofur treatment on the microbiome and its resistome, and possibly clarify the discrepancies observed in female pig 2, nanopore long-read shotgun sequencing was conducted, along with a targeted qPCR assay on the DNA of the fecal samples taken prior to the treatment and on the samples taken 9 h after the last administration of ceftiofur. High-molecular-weight total genomic DNA of fecal samples of ceftiofur-treated pigs was isolated using the QIAamp PowerFaecal Pro DNA Kit (QIAGEN, Antwerp, Belgium) according to the manufacturer’s instructions. Subsequently, DNA was quantified on a Nanodrop spectrophotometer and subjected to magnetic bead clean-up using CleanNGS (CleanNA, Waddinxveen, the Netherlands) whenever low-quality samples were observed. An amount of 400 ng DNA of each sample was used in the rapid barcoding library preparation (SQK-RBK110-96; ONT; Oxford, United Kindgom) as per manufacturer’s instructions. Final libraries, containing eight barcoded samples each, were loaded onto a R9.4.1 flow cell prior to sequencing on a GridION sequencer for 36 h. Raw data were collected in MinKNOW (v.21.05.12; ONT) and processed using the Guppy basecaller (-c dna_r9.4.1_450bps_supac.cfg) and demultiplexer (v. 5.0.12; ONT). Basecalled data were first quality filtered using NanoFilt (v.2.7.1; [77]) and subsequently used for taxonomic classification with Kraken2 (v.2.0.9; k2_pluspf_20210127; [78]). Based on this taxonomic classification, normalized OTUs of the 20 most abundant genera were determined using median N50 values and bacterial genome sizes, to correct for genera with larger genomes. Complete and subsampled (per genus) datasets were searched for antimicrobial resistance genes using Abricate (v.1.0.1; available online: https://github.com/tseemann/abricate (accessed on 13 August 2021) and CARD (updated on 15 June 2021; [79]).

Quantitative PCR was performed for five resistance genes, using the primers as described previously (Table 3) [80]. The reaction mixture consisted of PerfeCta SYBR Green FastMix (Quanta Bio, VWR, Leuven, Belgium) with 1 µL of DNA in a total volume of 10 µL. QPCR was performed on the qTOWER3 (Analytik Jena, Jena, Germany). Cycling conditions consisted of an initial enzyme activation step at 95 °C for 3 min, followed by 40 cycles of 15 s denaturation at 95 °C, 30 s of primer annealing at 60 °C and 30 s of elongation at 72 °C, followed by a final melt curve from 60 °C to 95 °C with 1 °C increment and 15 s of equilibration. The qPCR data were analyzed by the qPCRsoft software version 4.0 (Analytik Jena, Jena, Germany). Quantification cycles (Cq) were generated using the threshold cycle method, as generated by the software. The specificity of the primers was tested using melt curve analysis and gel electrophoresis of the PCR products. Ten-fold serial dilutions of pooled samples were used to assess the qPCR efficiency using robust regression [81]. Finally, the Cq values were analyzed in R using the SLqPCR package (version 1.56.0) [82] in R.

### 3.8. Statistical Analyses

For a comparative analysis of the microbiome between the ceftiofur fecal samples, the datasets were corrected to the relative total number of OTUs. The bacterial sequencing data, including the PCoA based on the Bray–Curtis dissimilarity matrix, were calculated by vegan in R package (V4.1.0) [83]. Permutational multivariate analysis of variance using distance matrices (adonis) was used to test for significance (vegan in R package, (number of permutations set to 1000)). A horizontal bar plot of the top 10 genera was visualized by using the R Graphics Package [84]. A Wilcoxon signed-ranks test (the R Stats Package [85]) was performed to evaluate the statistical changes in the relative total number of OTUs within the genera of interest. The changes of these genera of interest before and after treatment were visualized with box-and-whisker plots using the R Graphics Package. To correct for multiple testing, the Benjamini–Hochberg FDR correction was applied to determine the *q*-values in R.

For resistome comparison, the resistance gene counts from the long-read shotgun sequencing analysis were corrected to the relative base counts pre-treatment to post-treatment of the top 20 genera. A dependent Student’s t-test using SPSS 25.0 (IBM, Chicago, IL, USA) was performed to compare the corrected counts for the genes of interest before and after treatment.

For the qPCR data, a Wilcoxon signed-ranks test was used in R using the package stats (version 4.0.3).

## 4. Conclusions

The main aim of this study was to investigate the intestinal and fecal excretion of ceftiofur and cefquinome after intramuscular administration in pigs. Our findings indicate that both antimicrobials are excreted in the intestinal tract and are degraded at the cecal level. Cefquinome appears to be degraded more slowly in comparison to ceftiofur, since there is still a measurable concentration present in the cecum. Interestingly, one female pig excreted ceftiofur-related residues in all the collected fecal samples, suggesting no or only little degradation. For each pig from the ceftiofur group, a sample taken prior to ceftiofur treatment and one taken 9 h post-treatment was subjected to long-read nanopore shotgun sequencing and a targeted qPCR assay to determine whether the gut microbiome and its resistome played a role in this phenomenon. However, no clear microbiome or resistome discrepancies could be detected between pig n° 2 and the other pigs of this treatment group. It must be noted that the design of the current study was optimized as a pharmacokinetic study and was not intended for long-read nanopore shotgun sequencing and qPCR analysis. The limited sampling points post-treatment and the absence of a control group could pose challenges for comparative genome analysis. However, this hypothesis-free approach allowed for a general overview of the microbiome and provided information to design a thorough follow-up study. This future study will be designed in such a way as to overcome most of the current limitations (e.g., control group, no tazobactam addition, more sampling points after treatment cessation, etc.) and generate reliable and statistically significant results. Furthermore, it will focus on the difference in the impact of ceftiofur and cefquinome on the gut microbiome to provide well-founded advice on the choice of antimicrobial treatment to prevent the spread of antimicrobial resistance genes through the gut microbiome.

## Figures and Tables

**Table 1:** Overview of mean plasma pharmacokinetic (PK) parameters (±standard deviation): area under the curve from 0 to ∞ (AUC_0→__∞_) for al analytes, from 0 to 2 h (AUC_0→2 h_) for ceftiofur, from 0 to 24 h (AUC_0→24 h_) for desfuroylceftiofuracetamide and from 0 to 4 h (AUC_0→4 h_) for cefquinome, and calculated maximal plasma concentration (C_max_), time of C_max_ (T_max_) after the first administration, elimination half-life (T_1/2el_) within these time frames.

PK Parameter	Ceftiofur	Desfuroylceftiofuracetamide	Cefquinome
AUC_0→∞_ (h∗µg·L^−1^)	496 ± 142	85,260 ± 16,623	9445 ± 1118
AUC ^a^ (h∗µg·L^−1^)	474 ± 128	70,344 ± 13,238	8758 ± 1170
C_max_ (µg·L^−1^)	813 ± 177	9425 ± 2674	5547 ± 1030
T_max_ (h)	0.3 ± 0.1	0.8 ± 0.1	0.3 ± 0.2
T_1/2el_ (h)	0.4 ± 0.2	8.8 ± 1.8	1.2 ± 0.2

^a^ AUC_0→2 h_ for ceftiofur, AUC_0→24 h_ for desfuroylceftiofuracetamide and AUC_0→4 h_ for cefquinome.

**Table 2:** Intestinal content concentrations (mean ± standard deviation) of ceftiofur-related residues (measured as desfuroylceftiofuracetamide) and cefquinome in the different gastro-intestinal segments at 11 h after last intramuscular administration of 3 mg·kg^−1^ body weight ceftiofur or 2 mg·kg^−1^ body weight cefquinome.

Segment	Ceftiofur (Measured as Desfuroylceftiofuracetamide) (ng·g^−1^)	Cefquinome (ng·g^−1^)
Duodenum	50.2 ± 31.8 (*n* = 6)	17.5 ± 10.6 (*n* = 8)
Jejunum	57.4 ± 45.3 (*n* = 6)	38.2 ± 20.5 (*n* = 8)
Ileum	187.8 ± 101.7 (*n* = 7)	57.8 ± 37.5 (*n* = 8)
Cecum	<limit of quantification ^a^	6.4 ± 4.2 (*n* = 6)

^a^ Limit of quantification: 30 ng·g^−1^.

**Table 3:** Primers used in this study [80].

Target	Name	Sequence (5′–3′)
*bla*_TEM_	TEM-fwd TEM-rev	GCATCTTACGGATGGCATGA GTCCTCCGATCGTTGTCAGAA
*bla*_CMY_^a^	CMY-fwd CMY-rev	GGCAAACAGTGGCAGGGTAT AATGCGGCTTTATCCCTAACG
*bla*_SHV_	SHV-fwd SHV-rev	TCCCATGATGAGCACCTTTAAA TCCTGCTGGCGATAGTGGAT
*bla*_CTX m_^b,c^	CTX-A-fwd CTX-A-rev CTX-B-fwd CTX-B-rev	CGGGCRATGGCGCARAC TGCRCCGGTSGTATTGCC ACCGAGCCSACGCTCAA CCGCTGCCGGTTTTATC

^a^ *bla*CMY = *CIT-type AmpCs,* ^b^ *bla*CTX m A = *bla*CTX-M-1 and *bla*CTX-M-9, ^c^ *bla*CTX m B = *bla*CTX-M-2, *bla*CTX-M-8 and *bla*CTX-M-25.

## Data Availability

The raw data supporting the conclusions of this article are available from the corresponding author on reasonable request.

## Supplementary Materials

The following supporting information can be downloaded at: https://www.mdpi.com/article/10.3390/antibiotics11030342/s1. Figure S1: Genus-level horizontal bar plot of the relative total number of OTUs of the top 10 genera, Table S1: Mean resistance gene counts corrected to the relative base counts of the top 20 genera.
