# Supplementary material for: Intestinal Exposure to Ceftiofur and Cefquinome after Intramuscular Treatment and the Impact of Ceftiofur on the Pig Fecal Microbiome and Resistome
Source: Antibiotics (Basel). 2022 Mar 4;11(3):342. doi: 10.3390/antibiotics11030342 (PMC8944603; doi:10.3390/antibiotics11030342)
Supplement: Supplementary file 1 [file antibiotics-11-00342-s001.zip › antibiotics-1608312-supplementary.pdf]

Supplementary data

Intestinal exposure to ceftiofur and cefquinome after intramuscular treatment and the impact of ceftiofur on the pig faecal microbiome and resistome

Sofie Rutjens[1], Nick Vereecke[2], Ward De Spiegelaere[3], Siska Croubels[1], and Mathias Devreese[1,*]

[1] Department of Pathobiology, Pharmacology and Zoological Medicine, Faculty of Veterinary Medicine, Ghent University, 9820 Merelbeke, Belgium; Sofie.Rutjens@UGent.be, Siska.Croubels@UGent.be
[2] PathoSense BV, 2500 Lier, Belgium & Department of Translational Physiology, Infectiology and Public Health, Laboratory of Virology, Faculty of Veterinary Medicine, Ghent University, 9820 Merelbeke, Belgium; Nick.Vereecke@pathosense.com
[3] Department of Morphology, Imaging, Orthopedics, Rehabilitation and Nutrition, Faculty of Veterinary Medicine, Ghent University, 9820 Merelbeke, Belgium; Ward.DeSpiegelaere@UGent.be

* Correspondence: Mathias.Devreese@UGent.be; Tel.: 0032 9 264 73 47

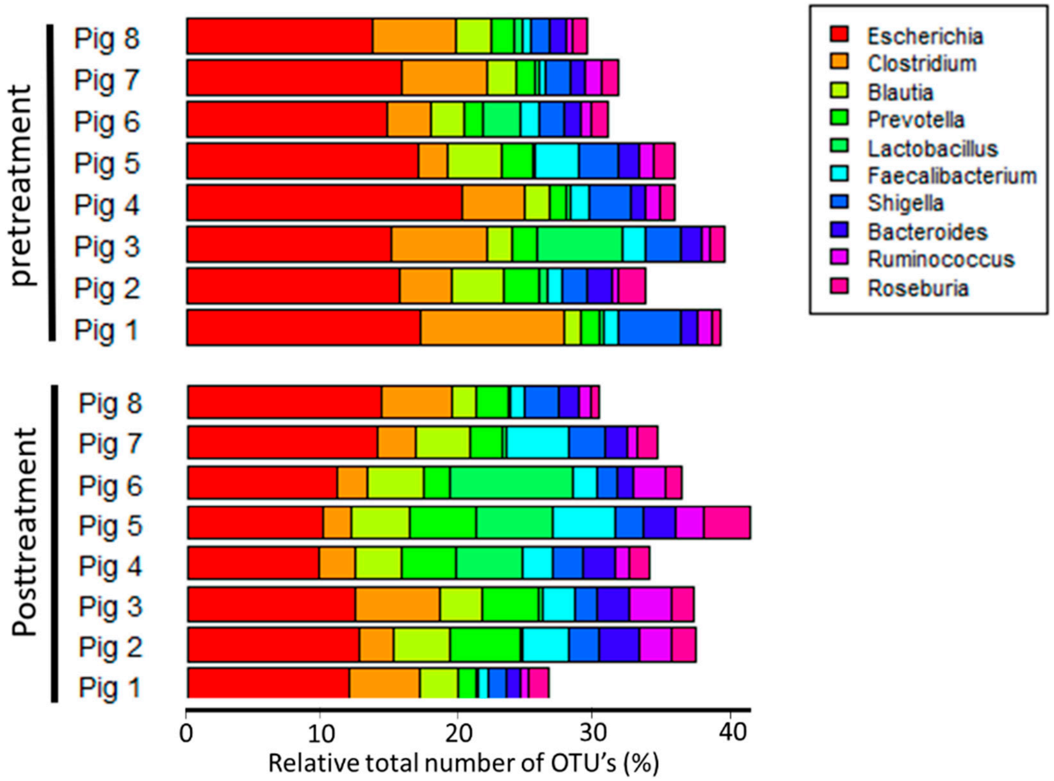

Figure S1. Genus level horizontal bar plot of the relative total number of OTU's of the top 10 genera. Faecal microbiomes for each animal (n=8) were analysed pre-treatment and 9 hours after the last dose of the daily intramuscular administration of 3 $mg.kg^{-1}$ body weight of ceftiofur for 3 consecutive days. Collection times and animal numbers are displayed on the side.

Table S1. Mean (± standard deviation, n=8) resistance gene counts corrected to the relative (pre-treatment to post-treatment, i.e. 9 hours after the last dose of the daily intramuscular administration of 3 $mg.kg^{-1}$ body weight of ceftiofur for 3 consecutive days) base counts of the top 20 genera. *Tet* = tetracycline, *AME* = aminoglycoside-modifying enzyme, *Van* = vancomycine, *CfxA* = beta-lactamase.

Genus	*Tet* resistance genes		*AME* resistance genes		*VAN* resistance genes		Macrolide resistance genes		Lincosamide resistance genes		*CfxA* resistance genes	
	**Day 0**	**Day 3**	**Day 0**	**Day 3**	**Day 0**	**Day 3**	**Day 0**	**Day 3**	**Day 0**	**Day 3**	**Day 0**	**Day 3**
Anaerobutyricum	2.1 ± 2.1	2.2 ± 2.5	0.1 ± 0.4	0.1 ± 0.2	0	0	0.4 ± 0.7	0.1 ± 0.2	0.6 ± 1.2	0	0	0
Bacillus	0	0	0.3 ± 0.5	0.2 ± 0.5	0	0	0	0.3 ± 0.6	0.8 ± 2.1	0	0	0
Bacteroides	8.3 ± 6.6	7.8 ± 6.6	0	0	0	0	9.5 ± 7.3	5.9 ± 2.8	2.0 ± 4.7	0.2 ± 0.4	4.7 ± 2.5	7.6 ± 6.4
Blautia	0.5 ± 1.1	1.2 ± 1.1	0.3 ± 0.5	0.2 ± 0.4	0	0	0	0.1 ± 0.2	0.4 ± 0.5	0.2 ± 0.5	0	0
Clostridium	6.8 ± 3.5	9.8 ± 7.9	0.6 ± 0.7	0*	0	0	0.9 ± 1.0	2.4 ± 3.5	0.6 ± 1.4	2.8 ± 5.0	0	0
Escherichia	0	0	0.1 ± 0.4	0.7 ± 1.0	0	0	0.1 ± 0.4	0.1 ± 0.4	0.6 ± 0.5	0.7 ± 0.8	0	0
Faecalibacterium	7.8 ± 6.0	6.8 ± 3.9	0	0.1 ± 0.2	0	0	0.1 ± 0.4	0	0.6 ± 0.7	0.6 ± 0.5	0	0
Lachnoclostridium	1.9 ± 2.0	1.4 ± 1.0	0.1 ± 0.4	0.1 ± 0.3	0	0	0.3 ± 0.5	0.1 ± 0.3	0.1 ± 0.4	0.2 ± 0.4	0	0
Lactobacillus	3.3 ± 4.8	9.7 ± 19.3	0	0	0	0	2.1 ± 4.8	0.3 ± 0.6	5.0 ± 3.2	7.9 ± 19.6	0	0
Leishmania	0	0	0	0	0	0	0	0	0	0	0	0
Methanobrevibacter	0	0	0	0	0	0	0	0	0	0	0	0
Oscillibacter	1.4 ± 1.8	3.2 ± 3.8	0.1 ± 0.4	0	0	0	0	0	0.4 ± 0.5	0	0	0
Prevotella	0	0.1 ± 0.2	0	0	0	0	0	0	0	0	2.8 ± 2.0	0.7 ± 0.7*
Romboutsia	0.1 ± 0.4	0	0.1 ± 0.4	0	0	0	0.3 ± 0.5	0	0	0	0	0
Roseburia	2.5 ± 2.2	3.2 ± 2.0	0.3 ± 0.7	0.2 ± 0.3	0	0	0.8 ± 0.7	0.9 ± 1.0	0.1 ± 0.4	0.6 ± 0.9	0	0
Ruminococcus	0.5 ± 0.8	0.4 ± 0.8	0.3 ± 0.5	0	0	0	0	0.3 ± 0.6	0.2 ± 0.5	0.2 ± 0.4	0	0
Salmonella	0	0	0.5 ± 0.5	0.1 ± 0.3	0	0	0	0	0	0	0	0
Shigella	0	0	0	0	0	0	0	0	0.6 ± 1.4	0	0	0
Streptococcus	7.9 ± 5.7	13.3 ± 7.0	0.3 ± 0.5	0.1 ± 0.2	1.6 ± 1.6	1.2 ± 1.4	0.3 ± 0.7	0.5 ± 0.5	2.5 ± 3.3	1.5 ± 1.3	0	0
Treponema	0.1 ± 0.4	0	0	0	0	0	0	0	0.8 ± 1.2	0.6 ± 1.6	0	0

Significance codes: '*' *p*-value <0.05
